# Associations between breakfast skipping and outcomes in neuropsychiatric disorders, cognitive performance, and frailty: a Mendelian randomization study

**DOI:** 10.1186/s12888-024-05723-1

**Published:** 2024-04-02

**Authors:** Zheng Zhang, Jinglan Tan, Qinghua Luo

**Affiliations:** Department of Psychiatry, The First Affiliated Hospital of Chongqing Medical University, Chongqing, China

**Keywords:** Breakfast skipping, Attention deficit hyperactivity disorder, Major depressive disorder, Cognitive, Frailty, Mendelian randomization

## Abstract

**Background:**

Prior studies have identified a correlation between breakfast skipping and a heightened risk of mental health issues. This investigation aimed to employ a Two-Sample Mendelian Randomization (MR) approach to explore the potential causal links between breakfast skipping and various psychiatric, neurological disorders, cognitive performance, and frailty.

**Methods:**

Utilizing data from genome-wide association studies within European demographics, this research scrutinized the association between breakfast habits and several neuropsychiatric conditions and physical health outcomes, including Alzheimer’s disease (AD), Attention Deficit Hyperactivity Disorder (ADHD), Bipolar Disorder (BD), Major Depressive Disorder (MDD), Narcolepsy, Insomnia, cognitive performance, and frailty. In this MR analysis, the Inverse Variance Weighted (IVW) method was primarily utilized for evaluation. Outcomes were reported as Odds Ratios (*OR*) and regression coefficients (*β*), and underwent validation through False Discovery Rate (FDR) corrections, thereby offering a rigorous evaluation of the effects of breakfast habits on both mental and physical health dimensions.

**Results:**

Findings demonstrate a significant causal link between skipping breakfast and an increased risk of ADHD (*OR* = 2.74, 95%CI: 1.54–4.88, *P*_FDR_ = 0.003) and MDD (*OR* = 1.7, 95%CI: 1.22–2.37, *P*_FDR_ = 0.005). Conversely, no substantial causal associations were identified between breakfast skipping and AD, BD, narcolepsy, or insomnia (*P*_FDR_ > 0.05). Moreover, a notable causal relationship was established between skipping breakfast and a reduction in cognitive performance (*β* = -0.16, 95%CI: -0.29–0.04, *P*_FDR_ = 0.024) and an increase in frailty (*β* = 0.29, 95%CI: 0.12–0.45, *P*_FDR_ = 0.003).

**Conclusion:**

The MR analysis reveals that skipping breakfast is associated with an increased risk of ADHD, MDD, decreased cognitive performance, and greater frailty, while showing no associations were found with AD, BD, narcolepsy, or insomnia. These findings warrant further investigation into the underlying mechanisms and emphasize the importance of regular breakfast consumption for mental and physical well-being.

**Supplementary Information:**

The online version contains supplementary material available at 10.1186/s12888-024-05723-1.

## Introduction

Breakfast, considered the most crucial meal due to its impact on body metabolism and circadian rhythm, is often skipped in today's society [1], leading to various health implications. Research indicates that skipping breakfast is a growing trend with significant links to an increased risk of overweight, obesity, cardiovascular diseases, and type 2 diabetes [2]. A notable cross-sectional study, involving 21,972 individuals from 28 countries, demonstrated an association between breakfast skipping and poor psychiatric health as well as diminished academic performance [3]. Furthermore, a Brazilian study highlighted that daily breakfast consumption could reduce the incidence of common mental disorders, primarily encompassing depressive and anxiety disorders [4].

Currently, psychiatric disorders, as the leading cause of disability globally, pose a significant public health challenge, with adult lifetime prevalence rates ranging from 12.2% to 48.6% [5]. These disorders, including major depressive disorder (MDD), bipolar disorder (BD), anxiety and stress-related disorders (ASRDs), and schizophrenia, contribute substantially to suicide risks, with psychiatric conditions being implicated in approximately 70% of suicide cases [6]. The World Health Organization reports that around 280 million individuals suffer from MDD, making it the foremost cause of mental health-related disability worldwide [7, 8]. In south China, irregular meal patterns, particularly breakfast skipping, have been positively associated with an increased risk of MDD (Odds Ratio [OR] = 2.74; 95% Confidence Interval [CI]: 1.73–4.33) [9]. Additionally, individuals with BD often exhibit unhealthy eating behaviors, including meal skipping, which in those with an evening chronotype, is linked to higher body mass indexes (BMIs) and metabolic disturbances [10].

Attention Deficit Hyperactivity Disorder (ADHD), a common neurodevelopmental disorder, manifests through persistent patterns of inattention, hyperactivity, and impulsivity, significantly affecting cognitive, academic, and social functions [11]. With advancing age, individuals are prone to neurodegeneration, leading to mild cognitive impairment that may progress to severe cognitive impairment, or Alzheimer’s disease (AD) [12], which are highly prevalent, affecting nearly 50 million people worldwide [13]. Previous studies suggest breakfast consumption may temporarily enhance cognitive performance, potentially due to improvements in blood glucose levels, neurotransmitter activity, and hormonal balances [14]. Dietary factors have also been implicated in neurological conditions, with the ketogenic diet showing potential in improving cognitive functions and attenuating AD neuropathology [15]. A case–control study of 360 children indicated that adherence to a Mediterranean diet, rich in vegetables, legumes, fruits, nuts, grains, and fish, was associated with reduced odds of ADHD [16]. Yet, the causal relationships between breakfast habits and these neuropsychiatric conditions remain underexplored.

In addition to diet, another crucial yet often overlooked factor in mental and cognitive health is sleep. Sleep significantly correlates with emotional states, and its quality is closely linked to depressive symptoms [17], increased stress [18], and altered alertness and mood regulation [19]. Insomnia, the most prevalent sleep disorder among both the general population and in clinical settings, affects 19%–50% of adults, with 10%–15% enduring chronic insomnia [20]. Remarkably, sleep disturbances may be intricately linked to breakfast habits. Research from Iran has demonstrated that individuals who seldom or never consume breakfast exhibit a higher probability of insomnia compared to those who consistently eat breakfast (Odds Ratio [OR]: 0.56, 95% Confidence Interval [CI]: 0.36–0.88) [21]. Despite this, many studies have yet to incorporate sleep adjustments in their analyses or investigate the potential contribution of breakfast skipping to sleep-related problems, such as narcolepsy [22]. Furthermore, frailty—a syndrome especially prevalent among the elderly and characterized by increased susceptibility to minor stressors and significant functional decline—has been shown to have substantial overlap with psychiatric disorders [23–25]. Systematic reviews and meta-analyses suggest that individuals with psychiatric conditions experience significantly greater cognitive difficulties than their healthier counterparts [26]. While breakfast can offer a short-term enhancement in cognitive performance through various biological mechanisms [14], the long-term implications of breakfast habits on cognition, frailty, and psychiatric conditions warrant further investigation.

Previous research has suggested links between breakfast skipping and multiple neuropsychiatric disorders, yet these findings warrant cautious interpretation due to potential confounding factors and the risk of reverse causation. In contrast to traditional observational studies prone to such biases, MR employs genetic variation as an instrumental variable, offering a refined method to establish causal relationships. This study uses MR to explore the associations between breakfast skipping and neuropsychiatric conditions such as MDD, BD, AD, ADHD, Narcolepsy, and Insomnia, as well as their impacts on cognitive function and frailty. Through this analysis, we aim to contribute to the broader comprehension of the implications of breakfast consumption patterns on mental health outcomes.

## Materials and methods

### Study design

**Fig. 1:**
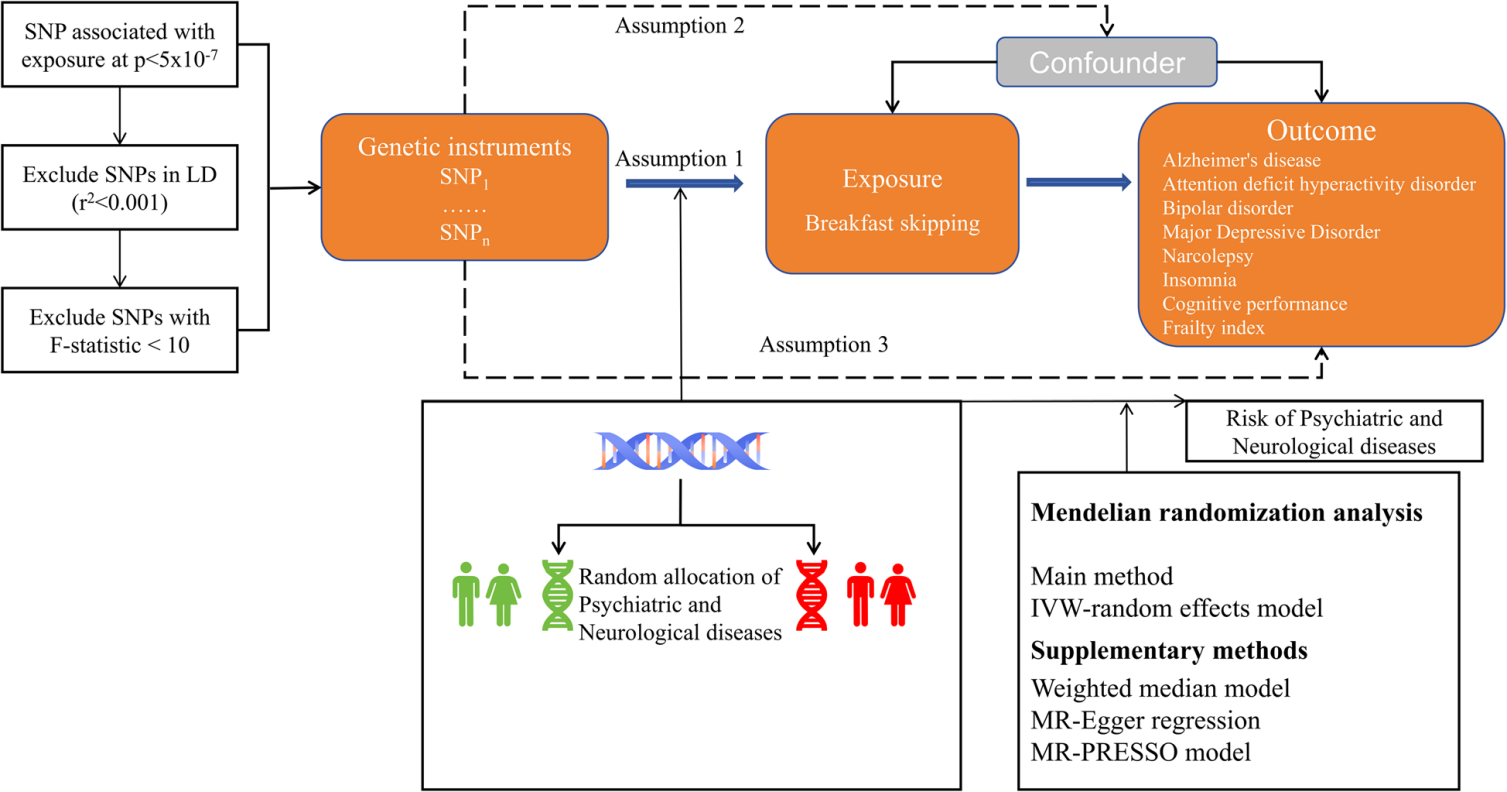
Schematic representation of the three hypotheses of the MR study. Solid arrow lines indicate MR analysis processes and can only influence the outcome by exposure. Dashed arrows indicate instrumental variables independent of any confounding variables. IVW: inverse-variance weighted; LD: linkage disequilibrium; SNP: single-nucleotide polymorphism

Our MR study leveraged published meta-analyses from Genome-Wide Association Studies (GWAS), data from the UK Biobank, pooled data from the Psychiatric Genomics Consortium (PGC), and datasets from the International Genomics of Alzheimer’s Project (IGAP). Single nucleotide polymorphisms (SNPs) were proxies for phenotypes and genetic instrumental variables (IVs) in the two-sample MR study. We screened the SNPs to ensure they met the three main MR hypotheses: assumption 1: a strong association with "skipping breakfast"; assumption 2: independence from potential confounders related to outcomes; assumption 3: no direct relationship between IVs and outcomes [27]. We used MR analysis to assess the causal relationship between breakfast skipping and neuropsychiatric disorders, cognitive performance, and frailty. Additionally, the process is illustrated in Fig. 1.

## Data source

Pertaining to the dataset on "breakfast skipping", we selected the most comprehensive Genome-Wide Association Study (GWAS) with a sample size of 193,860, available for download from the TYPE 2 DIABETES KNOWLEDGE PORTAL (https://t2d.hugeamp.org/). The dietary data were sourced from these 193,860 participants, among whom 106,284 were females, making up approximately 54% of the total sample. In this study, dietary data from 211,036 participants were collected using the Oxford WebQ, a web-based 24-h diet recall tool. Participants, with an average age of 56.8 years and a BMI of 26.9 kg/m^2^, reported their dietary intake over the past 24 hours [28]. Breakfast cereal skipping was assessed through up to 5 web-based 24-h diet recalls, categorizing responses as "breakfast skipping" (always responded "No"), "sometimes breakfast skipping" (occasionally responded "Yes"), and "breakfast consumers" (always responded "Yes") [29].

The datasets related to neuropsychiatric disorders comprise GWAS summary statistics from diverse sources. Specifically, the AD dataset includes 21,982 cases and 41,944 controls from IGAP, with diagnoses conforming to NINCDS-ADRDA or DSM-IV criteria [30]. The ADHD dataset, provided by the Integrative Psychiatric Research (iPSYCH) initiative, consists of 20,183 cases and 35,191 controls, with diagnoses established by psychiatrists according to ICD-10 standards [31]. The BD [32] and MDD [33], both sourced from PGC, include 41,917 cases with 371,549 controls and 59,851 cases with 113,154 controls, respectively; diagnoses in these collections adhered to internationally recognized criteria such as DSM-IV, ICD-9, or ICD-10. The study was confined to GWAS data from individuals of European descent who had undergone an ethical review and provided informed consent. Furthermore, the datasets for narcolepsy and insomnia, which comprise sample sizes of 460,913 and 462,341 respectively, were derived from the UKB (https://www.ukbiobank.ac.uk/) as a component of a comprehensive study involving 500,000 participants aged between 40 and 69 [34]. Assessment of these conditions was based on self-reported data. For insomnia, participants were asked if they had difficulty falling asleep at night or woke up in the middle of the night, referring to their experiences over the previous four weeks to guide their responses. They were provided with four response options: 'never/rarely', 'sometimes', 'usually', or 'prefer not to answer'. Cases of insomnia were defined by responses indicating 'usually', while 'never/rarely' or 'sometimes' were categorized as control responses. Narcolepsy was evaluated through questions about the likelihood of dozing off or falling asleep unintentionally during the day, such as while working, reading, or driving, with answer choices of 'never/rarely', 'sometimes', 'often', and 'all of the time'. These responses were then classified into a dichotomous trait, with 'never/rarely' and 'sometimes' indicating lower likelihood and 'often' and 'all of the time' indicating higher likelihood of daytime sleepiness.

A compilation of SNPs related to cognitive performance was gathered from the UKB and Cognitive Genomics Consortium datasets [35], encompassing approximately 10 million genetic variations observed in 257,841 individuals predominantly of European ancestry. The participants in the GWAS primarily focused on evaluating the General Cognitive Factor, operationalized through educational achievement and various cognitive assessments. These assessments comprised a comprehensive array of cognitive domains, including digital symbol coding, digit span, word reading, semantic fluency, visual memory, vocabulary, speech memory, phoneme fluency, and footprint testing. The incorporation of such diverse cognitive measures facilitated a thorough exploration of individuals' cognitive abilities, spanning aspects such as memory, language, and processing speed.

Frailty-related SNPs were derived from an extensive GWAS meta-analysis involving European participants from the UK B (*n* = 164,610, aged 60 to 70 years old, 51.3% females) and TwinGene (*n* = 10,616, aged 41 to 87 years old, 52.5% females) [36]. Frailty was evaluated using the frailty index, which is based on the accumulation of 49 health deficits throughout the life course. This well-validated measure is widely employed in clinical practice [37]. Details of the GWAS dataset are elucidated in Table 1.

**Table 1:** Summary information of the GWAS database in the two-sample MR study

Phenotype	Data source	Sample size	nSNPs	Population	PMID/GWAS ID
Breakfast skipping	UKB	193,860	14,661,601	European	31,190,057
Alzheimer’s disease	IGAP	63,926	10,528,610	European	30,820,047
Attention deficit hyperactivity disorder	PGC	55,374	8,094,094	European	30,478,444
Bipolar disorder	PGC	413,466	13,413,244	European	34,002,096
Major depressive disorder	PGC	173,005	13,554,550	European	29,700,475
Narcolepsy	UBK	460,913	9,851,867	European	ukb-b-5776
Insomnia	UBK	462,341	9,851,867	European	ukb-b-3957
Cognitive performance	NA	257,841	10,066,414	European	30,038,396
Frailty index	NA	175,226	7,589,717	European	34,431,594

*UKB* UK Biobank, *IGAP* International Genomics of Alzheimer’s Project, *PGC* Psychiatric Genomics Consortium, *SNP* Single nucleotide polymorphism, *NA* Not Applicable

### Selection of instrumental variables

The SNPs selected to proxy for the breakfast skipping instrumental variables all met the genome-wide statistical significance threshold (*P* < 5 × 10^–7^) to satisfy Hypothesis 1 to obtain independent SNPs [38]. These SNPs were further confirmed uncorrelated by a distance cut-off of 10,000 kilobases apart and a correlation index *r*^2^ < 0.001 [39]. To further evaluate the strength of instrumental variables, the *F* statistic was calculated for each SNP, where instrumental variables with *F* < 10 (considered as weak IV) were excluded which was calculated as* F* = [(*N*-*k*-1)/*k*] × [*R*^2^/(1-*R*^2^)], *R*^2^ was calculated by the following formula *R*^2^ = 2 × (1-MAF) × MAF × (*β*/SD)^2^ [40], *N* in the formula represents the sample size of the selected dataset, *k* is the total number of SNPs selected for MR analysis,* β* is the estimated SNP effect on breakfast skipping, SD is the standard deviation of *β*, and MAF is the minor allele frequency [41].

### Removal of confounding and palindromic SNPs

To address the second assumption of Mendelian randomization, we thoroughly investigated each IVs and its proxied traits using the PhenoScannerV2 database (http://www.phenoscanner.medschl.cam.ac.uk/). IVs that claimed outcomes-relevant traits with an r^2^ threshold greater than 0.80 were removed [42]. To ensure consistency, we standardized the breakfast skipping and eight outcome datasets by eliminating all palindromic SNPs with intermediate allele frequencies from the selected instrumental SNP, Palindromic SNPs refer to SNPs whose alleles correspond to nucleotides that pair with each other in a DNA molecule, while intermediate allele frequencies denote allele frequencies between 0.01 and 0.30 [43].

### Effect statistics and sensitivity analysis

After compiling a list of SNPs based on selection criteria, we conducted an MR analysis to evaluate the overall effects of selected SNPs for breakfast skipping on neuropsychiatric disorders. To ensure accuracy, we utilized the inverse variance-weighted (IVW), MR-Egger, and weighted median (WM) methods, comparing effect estimates to validate the stability of the results [44]. The IVW method provided the most precise estimates, which assumes all SNPs are valid with no directional pleiotropy [45]. Results were reported in *OR* with a 95% CI. We also conducted heterogeneity assessment and sensitivity analyses to verify that heterogeneity and pleiotropy within the genetic instruments did not bias the MR results. Quantitative measures such as Cochran Q-statistics and I^2^-values were used to estimate heterogeneity between SNPs [46]. Additionally, we performed a "leave-one-out" sensitivity analysis by removing a different SNP in each iteration to assess the causal effect of outlying SNPs and guarantee that the MR estimates were not affected by their removal [47]. Lastly, we applied the Pleiotropy RESidual Sum and Outlier (PRESSO) analysis to identify and remove any pleiotropic outlying SNPs and provide outlier-adjusted estimates [48]. To enhance statistical rigor, we applied False Discovery Rate (FDR) correction to adjust for multiple comparisons, reducing the risk of type I errors. This adjustment ensures the significance and reliability of our findings across various hypotheses.

All statistical analyses were conducted using R statistical software with the “dev tools,” “TwoSampleMR,” “LDlinkR,” and “MRPRESSO” Packages (version 4.1.0, R Foundation for Statistical Computing, Vienna, Austria). All statistical tests were 2-sided, and the MR and sensitivity analyses' results regarding the causal effects of exposures and outcomes were considered statistically significant at *P* < 0.05.

## Results

### SNP screening

Following the screening criteria (*P* < 5 × 10^–7^, *r*^2^ < 0.001, *F* > 10) and excluding confounders potentially associated with the exposure, a total of 18 SNPs were included in our study as instrumental variables for breakfast skipping. Eight sets of instrumental variables were identified, aligning in the same direction as the AD, ADHD, BD, MDD, narcolepsy, insomnia, cognitive performance, and frailty datasets, excluding the palindromic SNPs. The SNPs exhibited robust statistical strength, with F-statistics ranging from 145.74 to 198.08, significantly exceeding the conventional threshold of 10, as shown in Table 2.

**Table 2:** Summary of sensitivity analysis results

Exposure & Outcome	MR-Egger regression		Cochran’s Q		MR-PRESSO	*F*	Power (%)
	Intercept	*P*	Q value	*P*	Global test *P* value		
Alzheimer’s disease	0.019	0.356	13.7	0.32	0.322	198.08	100
Attention deficit hyperactivity disorder	0.008	0.709	14.23	0.221	0.284	162.87	100
Bipolar disorder	0.006	0.741	8.2	0.769	0.764	171.3	35
Major depressive disorder	0.007	0.514	12.57	0.249	0.339	154.07	100
Narcolepsy	-0.003	0.116	22.53	0.032	0.062	161.62	80
Insomnia	-0.001	0.974	24.67	0.055	0.204	199.07	80
Cognitive performance	-0.001	0.694	13.43	0.201	0.238	145.74	88
Frailty index	-0.004	0.53	27.78	0.01	0.071	185.97	100

*MR-PRESSO* Sum of outliers and multiplicity residuals, *F* F-statistic, *Power* Statistical efficiency

### MR estimates of causal effect

**Fig. 2:**
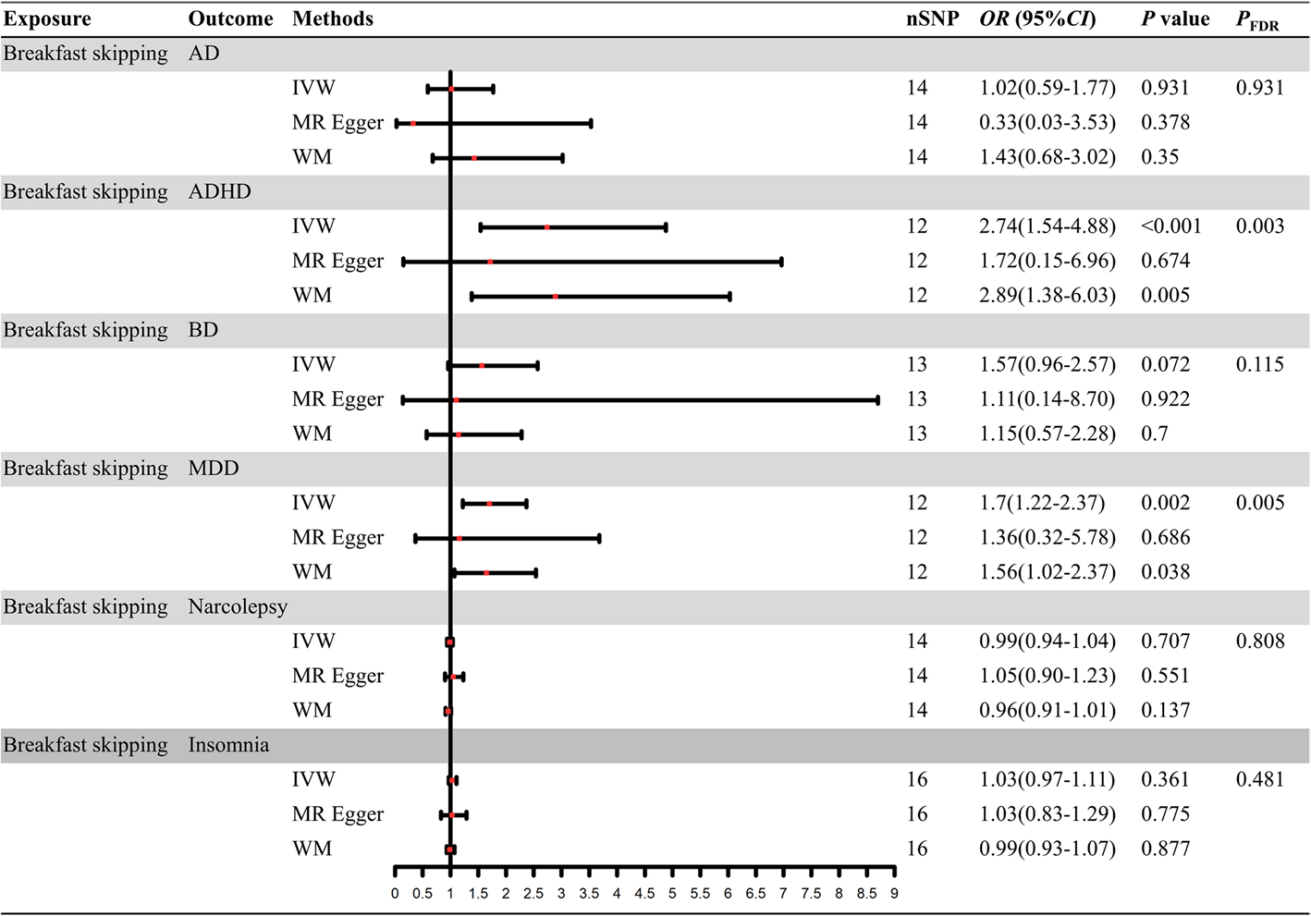
Forest plot of causal relationship between breakfast skipping and six neuropsychiatric disorders risk based on three MR methods

**Fig. 3:**
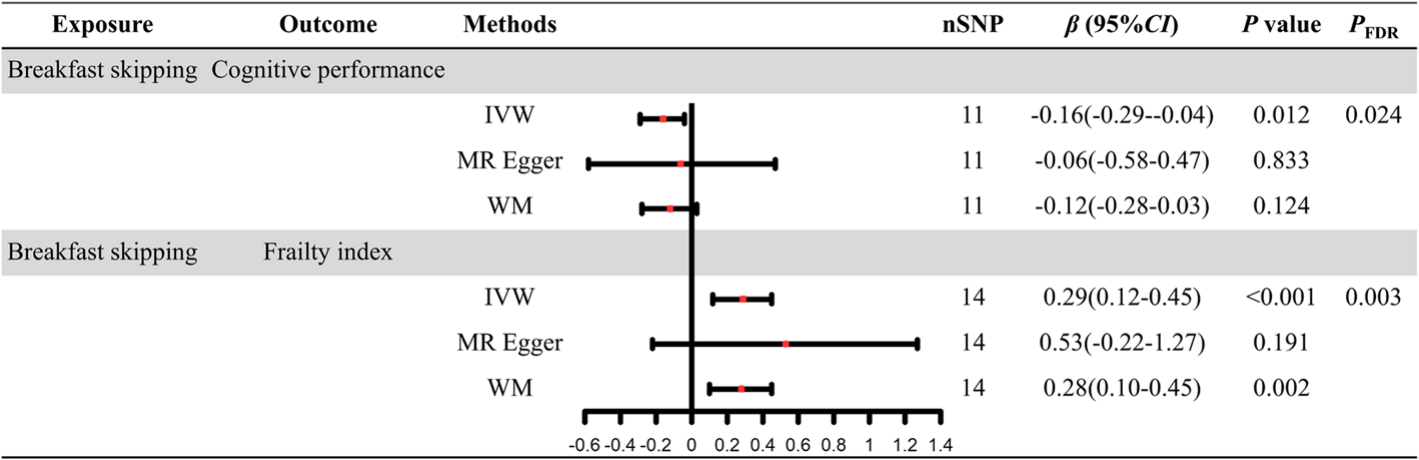
Forest plot of causal relationship between breakfast skipping and cognitive performance and frailty on three MR methods. AD: Alzheimer's disease; ADHD: attention deficit and hyperactivity disorder; BD: bipolar disorder; MDD: major depressive disorder; *OR*: odds ratio; *CI*: confidence interval; IVW: inverse variance weighting; WM: weighted median; MR Egger: MR Egger regression; nSNP: number of single-nucleotide polymorphism

As shown in Figs. 2 and 3, The IVW method revealed a potential causal impact of skipping breakfast on ADHD (IVW: *OR* = 2.74; 95% *CI*: 1.54–4.88;* P*_FDR_ = 0.003), MDD (IVW: *OR* = 1.7; 95% *CI*: 1.22–2.37; *P*_FDR_ = 0.005), cognitive performance (IVW: *β* = -0.16; 95% *CI*:—0.29–0.04; *P*_FDR_ = 0.024), and frailty (IVW: *β* = 0.29; 95% *CI*: 0.12–0.45; *P*_FDR_ = 0.003), These findings suggest that omitting breakfast may serve as a potential risk factor for both ADHD and MDD, in addition to leading to decreased cognitive performance and increased frailty. Furthermore, the IVW analysis results for AD (IVW: *OR* = 1.02; 95% *CI*: 0.59–1.77; *P*_FDR_ = 0.931), BD (IVW: *OR* = 1.57; 95% *CI*: 0.96–2.57; *P*_FDR_ = 0.115), and insomnia (IVW: *OR* = 1.03; 95% *CI*: 0.97–1.11; *P*_FDR_ = 0.481) were consistent with previous observations, suggesting that skipping breakfast could be a potential risk factor for these conditions, though these results did not achieve statistical significance. Additionally, the relationship between skipping breakfast and narcolepsy (IVW: *OR* = 0.99; 95% *CI*: 0.94–1.04; *P*_FDR_ = 0.707) was also found to be non-significant.

**Fig. 4:**
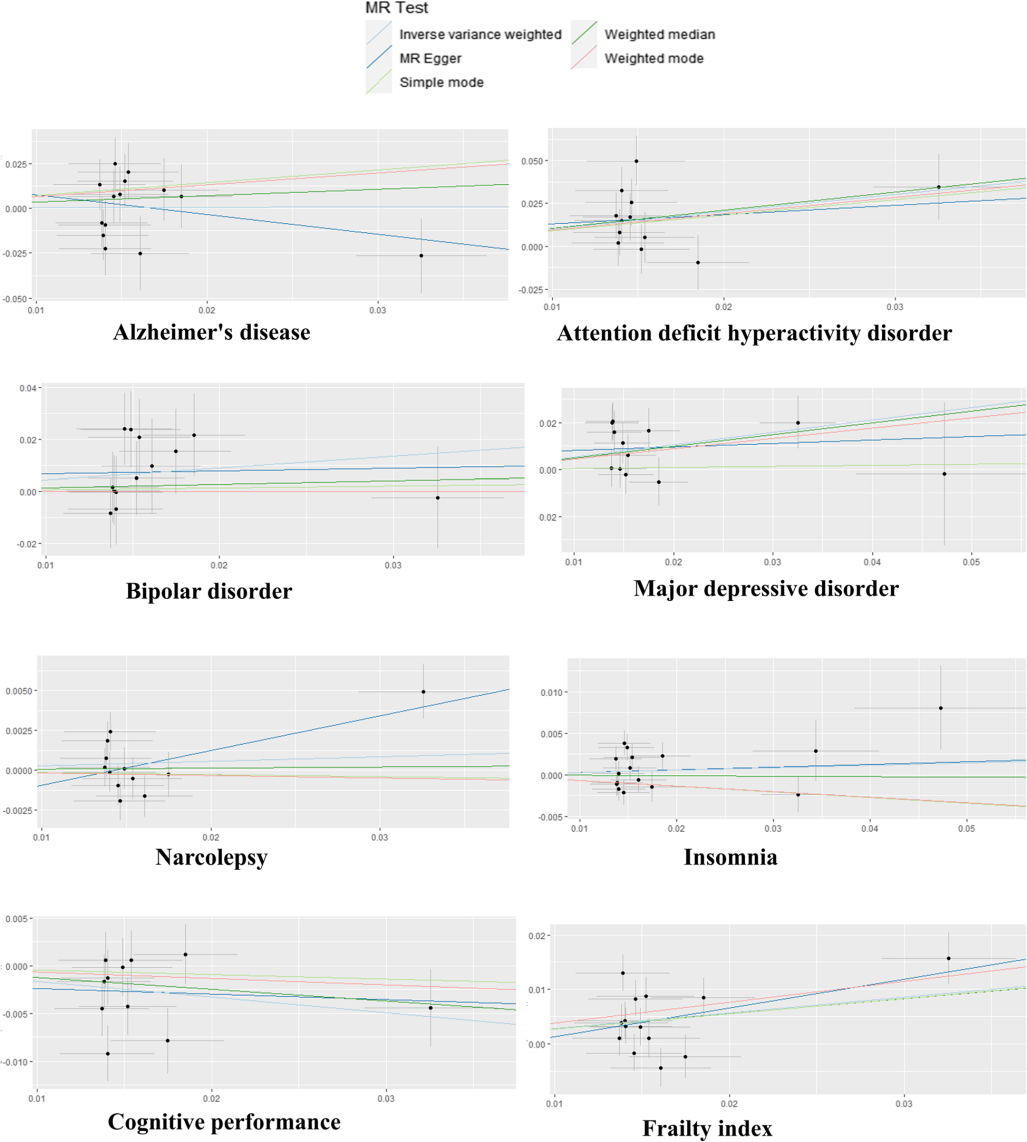
Scatter plot of causal relationship between breakfast skipping and neuropsychiatric disorders, cognitive performance, and frailty risk based on three MR methods. The horizontal x-axes indicate the genetic instruments linked to the exposure data, while the vertical y-axes represent the genetic instruments associated with the outcome data. The IVs employed in the MR analysis are indicated by black dots. Light blue: inverse-variance weighted; green: weighted-median estimator; deep blue: MR-Egger. As the inverse-variance weighted and weighted-median estimator methods produced highly similar estimates in the analysis, these figures exhibit a visual overlap

Figure 4 shows the scatterplots plotted by the five methods of MR.

### Heterogeneity and pleiotropy

The MR-Egger regression did not show any pleiotropy in the MR analyses for AD (Egger intercept = 0.019, *P* = 0.356), ADHD (Egger intercept = 0.008,* P* = 0.709), BD (Egger intercept = 0.006, *P* = 0.741), MDD (Egger intercept = 0.00, *P* = 0.514), narcolepsy (Egger intercept = -0.003, *P* = 0.116), insomnia (Egger intercept = -0.001, *P* = 0.974), cognitive performance (Egger intercept = -0.001, *P* = 0.694) and frailty (Egger intercept = -0.004, *P* = 0.53). In the Cochran's Q test for Narcolepsy, the results revealed a *P*-value of 0.032, indicating a certain degree of statistical heterogeneity. However, despite this heterogeneity, it does not significantly compromise the reliability of the IVW method structure. In addition, the MR-PRESSO test was performed, and consistent results were obtained, with none revealing the presence of pleiotropic and outlier SNPs, as shown in Table 2.

## Discussion

Our study employed the MR method to rigorously investigate the association between skipping breakfast and neuropsychiatric disorders, alongside cognitive performance and frailty. The analysis demonstrated a causal link between skipping breakfast and increased risk of ADHD and MDD, showing positive correlations for both conditions. However, no significant causal associations were identified for AD, BD, narcolepsy or insomnia. Additionally, our research found that skipping breakfast was causally related to reduced cognitive performance and increased frailty, indicating the broad impact of breakfast habits on both mental health and physical well-being.

Many studies have shown that breakfast is essential for psychiatric and physical health. Some meta-analyses of observational studies concluded that skipping breakfast might be associated with higher risk of diabetes [2], primary dysmenorrhea [49], cardiovascular disease [50], overweight and obesity. Significantly, a recent meta-analysis incorporating data from 14 studies and a combined sample size of 399,550 individuals revealed a notable positive correlation between the act of skipping breakfast and the odds ratio for depression (pooled *OR*: 1.39; 95% CI: 1.34–1.44). This finding is in concordance with the results of our investigation, which also demonstrated that the omission of breakfast is significantly associated with an elevated risk of developing depression. A preceding study delved into the association between breakfast consumption and its impact on depression, focusing on carbohydrate ingestion. The study proposed that as blood glucose levels decrease during nocturnal fasting, cortisol release occurs. Elevated cortisol levels are correlated with increased inflammatory cytokines, particularly those that attenuate serotonin, a neurotransmitter implicated in mood regulation and depression alleviation [18]. Therefore, skipping breakfast may be a potential factor that increases the risk of depression. On the contrary, the act of consuming breakfast may modulate metabolic responses during fasting conditions, ensuring a sustained supply of nutrients to the central nervous system. This could potentially influence nutrient intake and status [51]. Given the fundamental role of nutrition in central nervous system functioning, including the regulation of neurotransmitters such as serotonin and dopamine, breakfast consumption emerges as a factor that may contribute to the association between nutrition and depressive symptoms [52].

Studies have also shown that skipping breakfast is associated with an increased risk of ADHD [53], which supports our findings. However, the etiological model of the causal association between breakfast and ADHD remains unknown. Several potential reasons may explain this causal association. Previous studies have identified a link between ADHD and eating disturbances. The shared biological mechanism in ADHD and binge eating does not function typically, leading to the utilization of immediate rewards, such as altered eating behavior and risk-taking [54]. In addition, an unbalanced diet can lead to deficiencies in essential nutrients [54]. For instance, iron and zinc, which contribute to healthy neurocognitive and physical growth, are cofactors for dopamine and norepinephrine production, which play an essential role in the etiology of ADHD [55]. What’s more, Omega-3 fatty acids seem also to have a relationship with ADHD [56].

Previous studies examining the relationship between breakfast consumption and cognitive performance have primarily employed cross-sectional designs, which inherently limit the ability to establish causality. The findings from these studies have been mixed and, at times, contradictory. Some research has reported minimal or no significant impact of breakfast consumption on cognitive functions [57]. Conversely, an increasing body of literature suggests that breakfast positively influences various cognitive domains in adults, children, and adolescents [14, 58], corroborating our research results. The enhancement in cognitive functions post-breakfast might be attributed to the nutritional quality of the first meal of the day, particularly breakfasts rich in complex carbohydrates. These meals are thought to support cognitive performance throughout the morning, possibly mediated by factors such as the glycemic index (GI) or glycemic load (GL). Notably, while a meta-analysis indicated that GL does not impact cognition up to 119 min after consumption, benefits in immediate episodic memory were observed after 120 min with lower GL meals [58, 59]. Additionally, there is a documented association between skipping breakfast and reduced intake of essential nutrients [1], which has been linked to cognitive decline [58]. This nutritional deficit underscores the potential cognitive risks associated with omitting the morning meal.

A substantial body of epidemiological research has examined the possible link between the regular omission of breakfast and increased frailty. For instance, a cross-sectional survey involving 723 adults from communities in Chongqing found that consistent breakfast consumption significantly reduces the likelihood of frailty [60]. Furthermore, a study with 3,758 individuals aged 60 and over indicated that daily breakfast intake might act as a protective factor, lessening the risk of developing prefrailty and frailty, the study also highlighted that the adverse long-term impacts of omitting breakfast on cognitive functions could be attributed to its association with heightened cardiometabolic risks [61]. Crucially, individuals who habitually skip breakfast were found to have a higher frailty odds ratio compared to those who eat breakfast regularly. This distinction was further emphasized by their lower dietary intake and reduced nutrient density [62]. Consistent with these findings, our MR study identified a causal relationship between breakfast skipping and an increased risk of frailty. Nevertheless, to corroborate this hypothesis, further investigation through randomized clinical trials is imperative.

In our MR study, we investigated potential links between skipping breakfast and the incidence of AD, BD, insomnia, and narcolepsy. Unexpectedly, our data did not indicate significant associations between these variables. This lack of correlation might be due to unaccounted confounding factors or the limitations of the genetic instruments used, which could diminish our capacity to detect true associations. Nonetheless, our findings should not dismiss the potential benefits of breakfast for mental health and sleep quality. Future studies should further examine the impact of breakfast on mental health and sleep, considering individual differences and genetic predispositions. Additional research, particularly randomized controlled trials, is needed to establish clearer causal relationships. Despite the absence of a direct link in our findings, we stress the importance of a regular, balanced breakfast for overall health. Continued research in this area is vital to deepen our understanding of breakfast's role in health outcomes.

## Limitations

Our study has several limitations. Firstly, we used a lenient significance threshold (*P* < 5 × 10^−7^) to include more SNPs associated with "skipping breakfast," a practice aligning with some psychiatric genetic research when few significant SNPs are available [38]. While this broadens our genetic instrument base, it also increases the potential for false positives and relies on SNP data mostly from European descent, limiting the diversity representation. The lack of data stratification by gender and age in our dataset precluded detailed demographic analyses, hindering the assessment of varied impacts across different groups. This calls for future studies to incorporate stratified data for more nuanced insights. Furthermore, while including narcolepsy and insomnia to assess the effects of breakfast habits on sleep quantity, our study did not comprehensively address broader sleep adjustments due to dataset constraints. Future research should broaden sleep-related variables to thoroughly explore the link between breakfast habits and sleep.

Overall, addressing these limitations in future studies would provide a fuller understanding of the complex relationships between breakfast habits and various health outcomes.

## Conclusion

In conclusion, our study utilizing Two-Sample Mendelian Randomization analysis demonstrates a causal link between skipping breakfast and an increased risk of ADHD, MDD, reduced cognitive performance, and frailty. These findings highlight the significance of breakfast consumption for mental and physical health, although no causal relationship was found with AD, BD, narcolepsy or insomnia. Further research is necessary to understand the mechanisms behind these associations and to explore the benefits of regular breakfast consumption across diverse populations.

### Supplementary Information

**Supplementary Material 1.**

## Data Availability

The data used for analysis were obtained from published studies and public databases (ieu open gwas, https://gwas.mrcieu.ac.uk/datasets/ PGC: https://pgc.unc.edu/for-researchers/download-results/). All data generated during this study are included in this article and supplementary material. And the data that support the findings of this study are available from the corresponding author upon reasonable request.
