# Supplementary material for: Associations between breakfast skipping and outcomes in neuropsychiatric disorders, cognitive performance, and frailty: a Mendelian randomization study
Source: BMC Psychiatry. 2024 Apr 2;24:252. doi: 10.1186/s12888-024-05723-1 (PMC10988815; doi:10.1186/s12888-024-05723-1)

Table 1 SNPs used for MR analysis

| Breakfast skipping on AD | | | | | |
| --- | --- | --- | --- | --- | --- |
| SNP | effect_allele | other_allele | beta | se | Pvalue |
| rs10123348 | T | C | 0.0140465 | 0.00269281 | 1.90E-07 |
| rs10783243 | A | G | 0.0137171 | 0.00268422 | 2.80E-07 |
| rs136519 | T | C | 0.0145331 | 0.00272997 | 9.50E-08 |
| rs1461224 | T | G | -0.0139123 | 0.00268473 | 2.80E-07 |
| rs2232648 | T | C | 0.0148869 | 0.00287024 | 2.00E-07 |
| rs35107470 | G | A | 0.0185142 | 0.00295277 | 3.50E-10 |
| rs4410790 | C | T | 0.0152078 | 0.00278119 | 6.10E-08 |
| rs4797242 | A | C | -0.0153949 | 0.00292983 | 1.40E-07 |
| rs610758 | C | T | -0.0140288 | 0.00273 | 2.80E-07 |
| rs637174 | A | G | 0.0160872 | 0.00286341 | 1.80E-08 |
| rs6986473 | T | C | 0.0174778 | 0.00321128 | 4.20E-08 |
| rs8097544 | G | A | 0.0325517 | 0.00382144 | 1.80E-17 |
| rs9320747 | G | T | -0.0146306 | 0.00270045 | 6.90E-08 |
| rs953754 | A | G | 0.0138289 | 0.00268895 | 2.40E-07 |
| Breakfast skipping on ADHD | | | | | |
| rs10123348 | T | C | 0.0140465 | 0.00269281 | 1.90E-07 |
| rs10783243 | A | G | 0.0137171 | 0.00268422 | 2.80E-07 |
| rs136519 | T | C | 0.0145331 | 0.00272997 | 9.50E-08 |
| rs1461224 | T | G | -0.0139123 | 0.00268473 | 2.80E-07 |
| rs2232648 | T | C | 0.0148869 | 0.00287024 | 2.00E-07 |
| rs35107470 | G | A | 0.0185142 | 0.00295277 | 3.50E-10 |
| rs4410790 | C | T | 0.0152078 | 0.00278119 | 6.10E-08 |
| rs4797242 | A | C | -0.0153949 | 0.00292983 | 1.40E-07 |
| rs610758 | C | T | -0.0140288 | 0.00273 | 2.80E-07 |
| rs8097544 | G | A | 0.0325517 | 0.00382144 | 1.80E-17 |
| rs9320747 | G | T | -0.0146306 | 0.00270045 | 6.90E-08 |
| rs953754 | A | G | 0.0138289 | 0.00268895 | 2.40E-07 |
| Breakfast skipping on BD | | | | | |
| rs10123348 | T | C | 0.0140465 | 0.00269281 | 1.90E-07 |
| rs10783243 | A | G | 0.0137171 | 0.00268422 | 2.80E-07 |
| rs136519 | T | C | 0.0145331 | 0.00272997 | 9.50E-08 |
| rs1461224 | T | G | -0.0139123 | 0.00268473 | 2.80E-07 |
| rs2232648 | T | C | 0.0148869 | 0.00287024 | 2.00E-07 |
| rs35107470 | G | A | 0.0185142 | 0.00295277 | 3.50E-10 |
| rs4410790 | C | T | 0.0152078 | 0.00278119 | 6.10E-08 |
| rs4797242 | A | C | -0.0153949 | 0.00292983 | 1.40E-07 |
| rs610758 | C | T | -0.0140288 | 0.00273 | 2.80E-07 |
| rs637174 | A | G | 0.0160872 | 0.00286341 | 1.80E-08 |
| rs6986473 | T | C | 0.0174778 | 0.00321128 | 4.20E-08 |
| rs8097544 | G | A | 0.0325517 | 0.00382144 | 1.80E-17 |
| rs953754 | A | G | 0.0138289 | 0.00268895 | 2.40E-07 |
| Breakfast skipping on MDD | | | | | |
| rs10783243 | A | G | 0.0137171 | 0.00268422 | 2.80E-07 |
| rs136519 | T | C | 0.0145331 | 0.00272997 | 9.50E-08 |
| rs1461224 | T | G | -0.0139123 | 0.00268473 | 2.80E-07 |
| rs193084249 | G | A | -0.0472742 | 0.00891598 | 1.10E-07 |
| rs2232648 | T | C | 0.0148869 | 0.00287024 | 2.00E-07 |
| rs35107470 | G | A | 0.0185142 | 0.00295277 | 3.50E-10 |
| rs4410790 | C | T | 0.0152078 | 0.00278119 | 6.10E-08 |
| rs4797242 | A | C | -0.0153949 | 0.00292983 | 1.40E-07 |
| rs610758 | C | T | -0.0140288 | 0.00273 | 2.80E-07 |
| rs6986473 | T | C | 0.0174778 | 0.00321128 | 4.20E-08 |
| rs8097544 | G | A | 0.0325517 | 0.00382144 | 1.80E-17 |
| rs9320747 | G | T | -0.0146306 | 0.00270045 | 6.90E-08 |
| rs953754 | A | G | 0.0138289 | 0.00268895 | 2.40E-07 |
| Breakfast skipping on narcolepsy | | | | | |
| rs10783243 | A | G | 0.0137171 | 0.00268422 | 2.80E-07 |
| rs11570072 | G | A | 0.0181333 | 0.00358926 | 4.60E-07 |
| rs136519 | T | C | 0.0145331 | 0.00272997 | 9.50E-08 |
| rs1461224 | T | G | -0.0139123 | 0.00268473 | 2.80E-07 |
| rs193084249 | G | A | -0.0472742 | 0.00891598 | 1.10E-07 |
| rs2232648 | T | C | 0.0148869 | 0.00287024 | 2.00E-07 |
| rs4797242 | A | C | -0.0153949 | 0.00292983 | 1.40E-07 |
| rs610758 | C | T | -0.0140288 | 0.00273 | 2.80E-07 |
| rs637174 | A | G | 0.0160872 | 0.00286341 | 1.80E-08 |
| rs6986473 | T | C | 0.0174778 | 0.00321128 | 4.20E-08 |
| rs73046569 | A | G | -0.0343799 | 0.00653188 | 1.50E-07 |
| rs8097544 | G | A | 0.0325517 | 0.00382144 | 1.80E-17 |
| rs9320747 | G | T | -0.0146306 | 0.00270045 | 6.90E-08 |
| rs953754 | A | G | 0.0138289 | 0.00268895 | 2.40E-07 |
| Breakfast skipping on insomnia | | | | | |
| rs10123348 | T | C | 0.0140465 | 0.00269281 | 1.90E-07 |
| rs10783243 | A | G | 0.0137171 | 0.00268422 | 2.80E-07 |
| rs136519 | T | C | 0.0145331 | 0.00272997 | 9.50E-08 |
| rs1461224 | T | G | -0.0139123 | 0.00268473 | 2.80E-07 |
| rs193084249 | G | A | -0.0472742 | 0.00891598 | 1.10E-07 |
| rs2232648 | T | C | 0.0148869 | 0.00287024 | 2.00E-07 |
| rs35107470 | G | A | 0.0185142 | 0.00295277 | 3.50E-10 |
| rs4410790 | C | T | 0.0152078 | 0.00278119 | 6.10E-08 |
| rs4797242 | A | C | -0.0153949 | 0.00292983 | 1.40E-07 |
| rs610758 | C | T | -0.0140288 | 0.00273 | 2.80E-07 |
| rs637174 | A | G | 0.0160872 | 0.00286341 | 1.80E-08 |
| rs6986473 | T | C | 0.0174778 | 0.00321128 | 4.20E-08 |
| rs73046569 | A | G | -0.0343799 | 0.00653188 | 1.50E-07 |
| rs8097544 | G | A | 0.0325517 | 0.00382144 | 1.80E-17 |
| rs9320747 | G | T | -0.0146306 | 0.00270045 | 6.90E-08 |
| rs953754 | A | G | 0.0138289 | 0.00268895 | 2.40E-07 |
| Breakfast skipping on cognitive performance | | | | | |
| rs10123348 | T | C | 0.0140465 | 0.00269281 | 1.90E-07 |
| rs10783243 | A | G | 0.0137171 | 0.00268422 | 2.80E-07 |
| rs1461224 | T | G | -0.0139123 | 0.00268473 | 2.80E-07 |
| rs2232648 | T | C | 0.0148869 | 0.00287024 | 2.00E-07 |
| rs35107470 | G | A | 0.0185142 | 0.00295277 | 3.50E-10 |
| rs4410790 | C | T | 0.0152078 | 0.00278119 | 6.10E-08 |
| rs4797242 | A | C | -0.0153949 | 0.00292983 | 1.40E-07 |
| rs610758 | C | T | -0.0140288 | 0.00273 | 2.80E-07 |
| rs6986473 | T | C | 0.0174778 | 0.00321128 | 4.20E-08 |
| rs8097544 | G | A | 0.0325517 | 0.00382144 | 1.80E-17 |
| rs953754 | A | G | 0.0138289 | 0.00268895 | 2.40E-07 |
| Breakfast skipping on frailty | | | | | |
| rs10123348 | T | C | 0.0140465 | 0.00269281 | 1.90E-07 |
| rs10783243 | A | G | 0.0137171 | 0.00268422 | 2.80E-07 |
| rs136519 | T | C | 0.0145331 | 0.00272997 | 9.50E-08 |
| rs1461224 | T | G | -0.0139123 | 0.00268473 | 2.80E-07 |
| rs2232648 | T | C | 0.0148869 | 0.00287024 | 2.00E-07 |
| rs35107470 | G | A | 0.0185142 | 0.00295277 | 3.50E-10 |
| rs4410790 | C | T | 0.0152078 | 0.00278119 | 6.10E-08 |
| rs4797242 | A | C | -0.0153949 | 0.00292983 | 1.40E-07 |
| rs610758 | C | T | -0.0140288 | 0.00273 | 2.80E-07 |
| rs637174 | A | G | 0.0160872 | 0.00286341 | 1.80E-08 |
| rs6986473 | T | C | 0.0174778 | 0.00321128 | 4.20E-08 |
| rs8097544 | G | A | 0.0325517 | 0.00382144 | 1.80E-17 |
| rs9320747 | G | T | -0.0146306 | 0.00270045 | 6.90E-08 |
| rs953754 | A | G | 0.0138289 | 0.00268895 | 2.40E-07 |
|  |  |  |  |  |  |

Fig 1 Eight outcome datasets' forest map


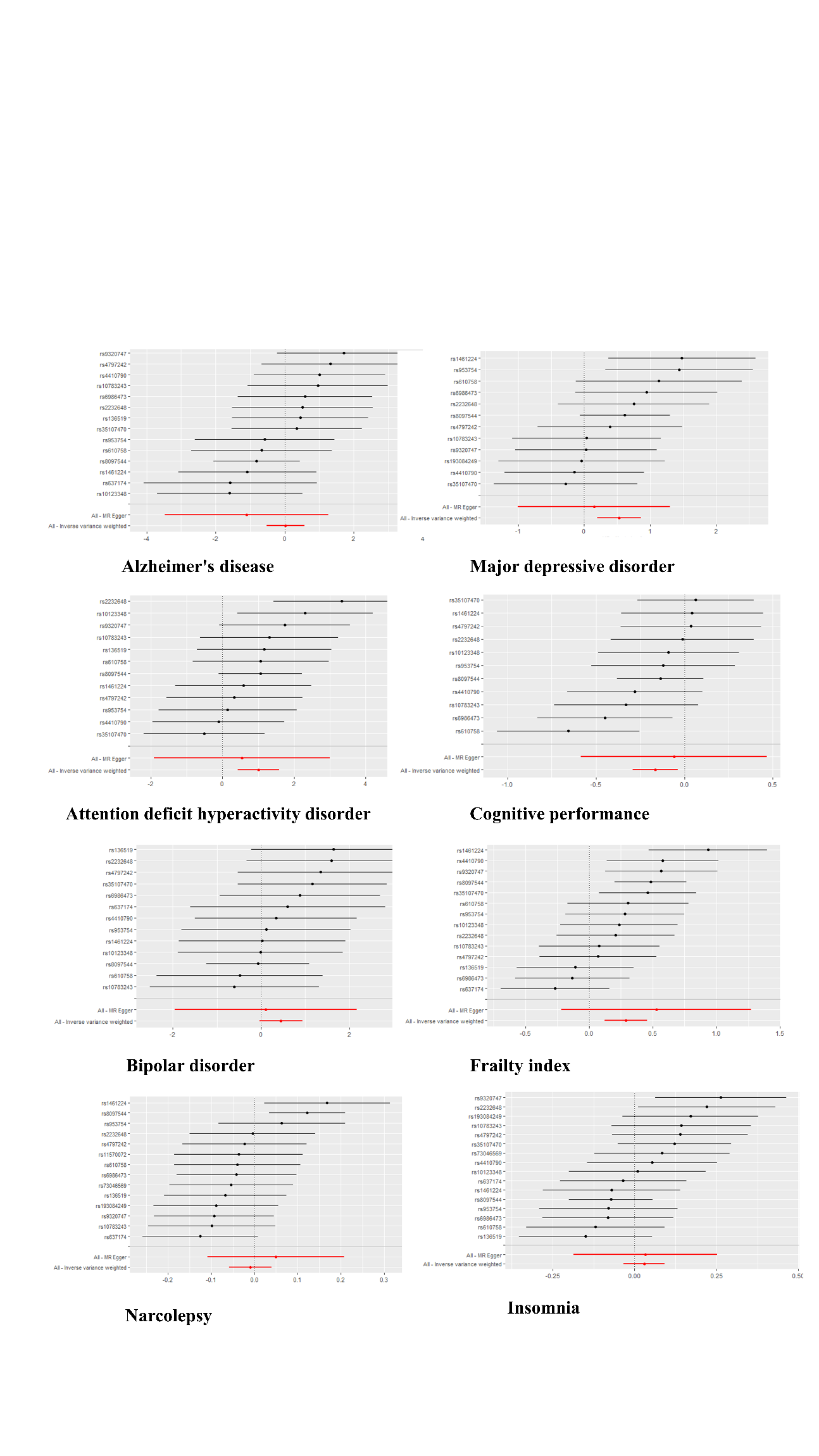


Fig 2 The Leave one method diagram of eight outcome datasets


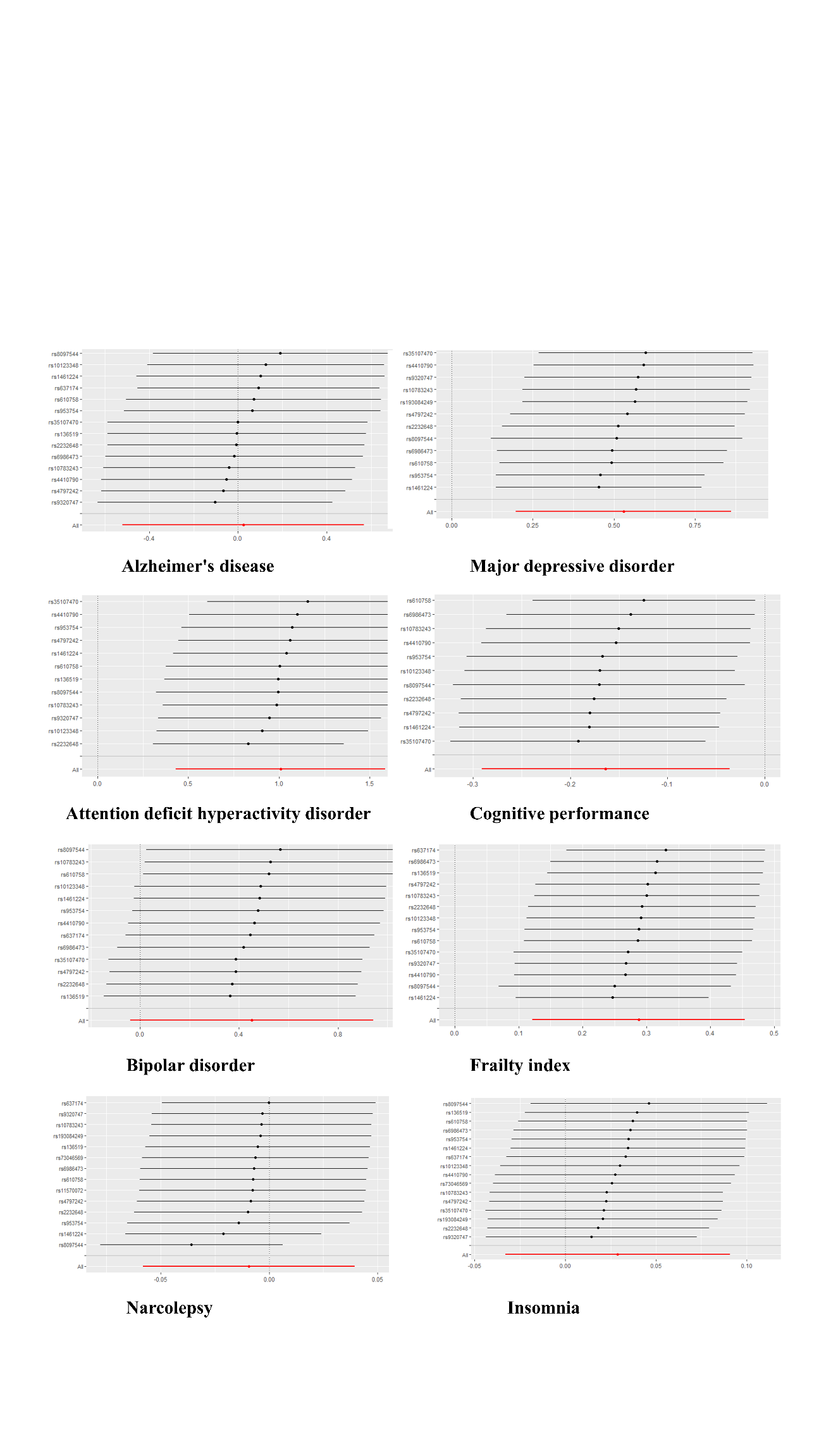

Supplement: Supplementary file 1 — Supplementary Material 1. [file 12888_2024_5723_MOESM1_ESM.docx]
